# Molecular Mechanism Pathways of Natural Compounds for the Treatment of Non-Alcoholic Fatty Liver Disease

**DOI:** 10.3390/molecules28155645

**Published:** 2023-07-25

**Authors:** Xiaolei Fang, Jiayu Song, Kaixuan Zhou, Xue Zi, Bin Sun, Huiwei Bao, Lijing Li

**Affiliations:** College of Pharmacy, Changchun University of Chinese Medicine, Changchun 130117, China; fangxl0927@163.com (X.F.);

**Keywords:** non-alcoholic fatty liver disease, natural compounds, oxidative stress, inflammation, hepatic steatosis

## Abstract

Non-alcoholic fatty liver disease (NAFLD) is the most common chronic liver disease in the world, and its incidence continues to increase each year. Yet, there is still no definitive drug that can stop its development. This review focuses mainly on lipotoxicity, oxidative stress, inflammation, and intestinal flora dysbiosis to understand NAFLD’s pathogenesis. In this review, we used NCBI’s PubMed database for retrieval, integrating in vivo and in vitro experiments to reveal the therapeutic effects of natural compounds on NAFLD. We also reviewed the mechanisms by which the results of these experiments suggest that these compounds can protect the liver from damage by modulating inflammation, reducing oxidative stress, decreasing insulin resistance and lipid accumulation in the liver, and interacting with the intestinal microflora. The natural compounds discussed in these papers target a variety of pathways, such as the AMPK pathway and the TGF-β pathway, and have significant therapeutic effects. This review aims to provide new possible therapeutic lead compounds and references for the development of novel medications and the clinical treatment of NAFLD. It offers fresh perspectives on the development of natural compounds in preventing and treating NAFLD.

## 1. Introduction

Non-alcoholic fatty liver disease (NAFLD) is one of the most common liver diseases. It is a condition characterized by hepatic steatosis, which is considered a hepatic manifestation of insulin resistance and is closely related to metabolic syndrome. There are two types of NAFLD: non-alcoholic steatohepatitis (NASH) and non-alcoholic fatty liver (NAFL) [1,2,3]. NAFLD is now thought to be the most prevalent chronic liver disease worldwide, with an epidemiological incidence of approximately 25%, which is continually rising [4,5]. The pathogenesis of NAFLD involves various factors, such as the reprogramming of interactions between hepatic stellate cells, hepatocytes, and sinus endothelial cells, modifications to the hepatic immune environment, the remodeling of the liver’s microvascular and stromal microenvironment, and disturbances in the metabolic balance [6]. Recent research has shown that novel possibilities for the treatment of NAFLD include obeticholic acid (OCA), cenicriviroc (CVC), and belapectin (GR-MD-02) [7,8,9,10]. There are some restrictions of the aforementioned medications, such as OCA’s capacity to raise low-density lipoprotein cholesterol (LDL-C) levels, itching, and high drug costs, as well as GR-MD-02’s potential to harm gastrointestinal and renal function, even though clinical trials have demonstrated the drugs’ significant effects on non-alcoholic fatty liver disease [7,11].

In recent years, NAFLD has been treated with natural products as well as synthetic small-molecule compounds derived from natural chemical structures. In the available therapies for non-alcoholic fatty liver disease, substances including flavonoids, alkaloids, phenolic compounds, saponins, and others have obvious benefits. It is crucial to thoroughly research these compounds [12].

In this review, we carried out an exploratory search using the PubMed database from the National Center for Biotechnology Information (NCBI) of the United States of America (available at https://pubmed.ncbi.nlm.nih.gov/ (accessed on 11 January 2023)). This platform comprises approximately 30 million citations of MEDLINE’s biomedical literature, life science journals, and online books. We searched the PubMed database and Google Scholar using the keywords “(Liver disease) OR (Nonalcoholic fatty liver disease)” and found a recently published article on the treatment of NAFLD with a natural compound. We then conducted a search using the terms “((Liver disease) OR (Nonalcoholic fatty liver disease)) AND (compound)” to find all studies on the treatment of NAFLD with this compound. We selected articles involving research using in-depth mechanisms for review.

In this review, we aimed to provide updated and comprehensive insights into in vitro and in vivo experiments involving natural compounds for the treatment of NAFLD and emphasize their potential mechanisms and molecular targets. This was done to provide novel potential leads and references for the development of innovative drugs for the treatment and management of non-alcoholic fatty liver disease.

## 2. Pathogenesis of NAFLD

Non-alcoholic fatty liver disease (NAFLD) is a type of liver disease characterized by steatosis of more than 5% of hepatocytes in the absence of alcohol intake [13]. According to the classic “two strikes” theory, NAFLD is characterized by two steps of liver damage: steatosis of the liver and non-alcoholic steatohepatitis, which is caused by inflammation of the liver [14]. However, it should be noted that the “two strikes” view has been updated. According to the “multiple strikes” hypothesis, a variety of wounds contribute to NAFLD (lipotoxicity, mitochondrial dysfunction, activation of the inflammatory pathway, and an imbalance of the intestinal microbiota) [15].

Triglycerides (TG) are the main form of fat accumulated in the liver of NAFLD patients [16,17]. Free fatty acids in adipose tissue, new adipogenesis (DNL), and diet are the major sources of TG. In mitochondria, FFA is synthesized from acetyl coenzyme A (CoA) [18]. TG itself has no hepatotoxicity whatsoever. Lipotoxic damage is caused by excessive FFA, which is conducive to the production of lipotoxic metabolites (such as ceramide, diacylglycerol, lysophosphatidylcholine, and ROS) [19]. Moreover, another significant feature of NAFLD is insulin resistance. Fat production may be increased by insulin. Two transcription factors play a key role in DNL: sterol regulatory element binding protein 1 (SREBP-1) and carbohydrate response element binding protein (ChREBP). The former is considered to be the key regulator of fatty acid synthesis [20]. As a major isoform of SREBP-1, sterol regulatory element-binding protein 1c (SREBP1c) enhances the DNL of glucose and FFA flow to the liver via peripheral insulin resistance, inhibiting the β-oxidation of FFA via nuclear receptor peroxisome proliferator-activated receptor alpha (PPAR-α) and promoting hepatic lipid accumulation [21].

Reactive oxygen species (ROS) play a key role in hepatic stellate cell proliferation, migration, and liver fibrosis in NAFLD. The activation of NADPH oxidases (NOXs) is considered to contribute to liver damage, with NOX2 being the main isoform responsible for ROS production [22]. There is a high level of NOX4 expression in liver stellate cells, hepatocytes, and myofibroblasts, and it is a key mediator of HSC activation during hepatic fibrogenesis. Transforming growth factor beta (TGF-β) is a powerful regulator of collagen synthesis and α-SMA expression in liver fibroblasts [23]. NOX4 is activated by TGF-β and Smad3 during fibrosis, and the upregulation of TGF-β leads to increased levels of extracellular matrix (ECM) compounds and elevated levels of ALT and fibrosis. NOX4 mediates the production of ROS during HSC activation [24]. Oxidative stress can be caused by excessive production of ROS in the liver, which can directly inhibit the production of antioxidant enzymes in the liver, such as superoxide dismutase (SOD) or catalase (CAT). It may also result in the depletion of antioxidant molecules like glutathione (GSH) [25]. This promotes the development of NAFLD. CYP2E1 is an important cause of ROS overproduction. Insulin resistance increases CYP2E1 expression and activity. Despite the absence of substrates, ROS is produced from oxygen, producing toxic intermediates such as dicarboxylic acids [26,27,28]. There is growing evidence that ER stress plays a critical role in the development of NAFLD, This is because the ER is responsible for maintaining hepatic lipid homeostasis through the unfolded protein response pathway (UPR). In NAFLD, the activity of inositol-dependent enzyme 1 (IRE1), X-box binding protein 1 (XBP1), and activating transcription factor (ATF6) is inhibited, and key enzymes of the lipid homeostasis pathway are downregulated, leading to hepatic steatosis [29]. Furthermore, steatosis triggers the activation of NF-κB, which leads to the production of pro-inflammatory cytokines such as tumor necrosis factor (TNF-α), interleukin-6 (IL-6), and interleukin-1β (IL-1β), which mediate liver inflammation through activating macrophages and increasing the action of the suppressor of cytokine signaling factor 3 (SOCS3) [13]. TNF-α also plays a role in mitochondrial dysfunction, inducing mitochondrial swelling that can lead to membrane rupture and interference between respiratory chains I and III [30].

**Figure 1:**
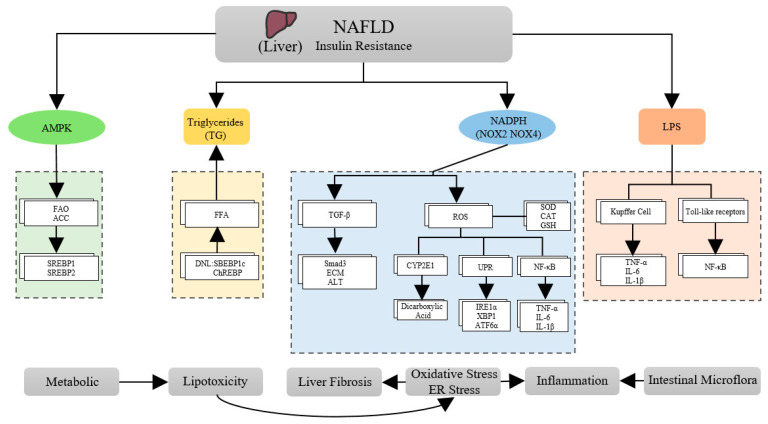
Some pathophysiological mechanisms of NAFLD.

In addition, dysbiosis of the gut microbiota is increasingly recognized as playing a significant role in the pathophysiology of NAFLD. Alterations in the gut microbiome increase intestinal permeability, and bacterial lipopolysaccharides (LPS) from Gram-negative bacteria result in excessive levels of reactive oxygen species (ROS) in the body. Additionally, LPS also upregulates TNF-α receptors in Kupffer cells, leading to liver inflammation. Additionally, some microorganisms produce endogenous ethanol, which increases the production of ROS in hepatic stellate cells and promotes the release of LPS from cells. Therefore, targeting the gut microbiome may be a promising therapeutic strategy for treating NAFLD [31]. It has been found that LPS translocation occurs when LPS is co-translocated with celiac particles, and the bacterial endotoxin is recognized by Toll-like receptors on hepatic stellate cells and KCs. This recognition activates the NF-κB pathway and subsequent inflammatory vesicles, leading to the development of inflammation and fibrosis in the liver [32]. AMPK inhibits the activity of key lipid synthesis enzymes and transcription factors such as fatty acid synthase and sterol regulatory element binding protein 1/2 (SREBP1/2). This inhibition results in decreased lipogenesis and increased fatty acid oxidation (FAO). Additionally, AMPK phosphorylates acetyl-CoA carboxylase (ACC), which inhibits its activity and results in decreased fatty acid synthesis and increased FAO [33] (Figure 1).

Non-alcoholic fatty liver disease (NAFLD) is a multifaceted disease microbiome resulting from a vicious cycle of lipotoxicity, oxidative stress, inflammation, and gut microbiome dysbiosis. These changes lead to complex alterations in liver histopathology and biochemistry. Insulin resistance is a key contributor to the development and progression of NAFLD. It exacerbates lipotoxicity, oxidative stress, and inflammation, ultimately leading to non-alcoholic steatohepatitis (NASH) [15]. It has been demonstrated that insulin resistance causes excessive liver fat buildup and increases oxidative stress. Moreover, it also leads to an excessive release of ROS and liver fibrosis, which can lead to mitochondrial dysfunction and the activation of NF-κB to trigger the expression of several genes, including pro-inflammatory mediators, resulting in inflammation of the liver. This implies that the “multi-hit” factors that have a cumulative effect on the liver are responsible for the development of NAFLD.

## 3. Natural Compounds

### 3.1. Flavonoid

Flavonoids are a group of compounds that are naturally present in many plants and are derived from 2-phenyl chromones as their parent nucleus structure [34]. Research has shown that flavonoids possess anti-inflammatory, anticancer, anti-aging, and antioxidant properties and can be used in a wide range of contemporary research [35,36,37,38]. In the treatment of NAFLD, flavonoids primarily exert their effects by reducing inflammation and oxidative stress.

#### 3.1.1. Quercetin

Quercetin is a natural flavonol compound, which widely exists in flavonoids in plants. It possesses several pharmacological properties, including antioxidant activity against free radicals, antibacterial properties, and cancer-fighting properties. Acacia rice is a rich source of quercetin and contains a high amount of this compound [39]. Quercetin is a flavonoid with potent oxidative properties and many studies have shown that it can significantly reduce lipid accumulation and SREBP1c and XBP-1 expression in adipocytes. It also acts on the AMPK pathways to inhibit the DNL pathway, thus exerting a direct anti-lipogenic effect [13,40]. Quercetin may be able to reduce the inflammatory response, oxidative stress, and abnormal lipid metabolism in patients with type 2 diabetes [41]. Several studies have demonstrated that quercetin can restore gut microbiota imbalances and activate the Toll-like receptor 4 (TLR-4) pathway, thereby reducing inflammatory responses, and decreasing hepatic steatosis caused by the accumulation of hepatic triglycerides [42,43]. Li et al. found that quercetin improved insulin resistance and reduced hepatic lipid accumulation by inhibiting the expression of the adipogenic genes SREBP1c and FAS in free fatty acid (FFA) and insulin-induced NAFLD HepG2 cell models, suggesting its potential as a treatment for NAFLD [44]. Quercetin also reduces hepatic steatosis through its antioxidant and anti-inflammatory activities, the inhibition of cell proliferation and apoptosis, and the inhibition of hepatic inflammatory responses through NF-κB and TLR inhibition, as well as the reduction of oxidative stress mediated by phosphatidylinositol 3-kinase/nuclear factor erythroid 2-related factor 2 (PI3k/Nrf2) [45]. Quercetin significantly increased anti-glutathione peroxidase four (GPX4) protein expression and decreased anti-cyclooxygenase 2 (COX2) and acyl-coenzyme A synthase long-chain family member four (ACSL4) protein expression, which suggests quercetin inhibits iron sagging in hepatocytes, as was demonstrated in NAFLD mice [46]. Quercetin also improves mitochondrial respiration and prevents obesity-induced hepatic lipid degeneration by boosting heme oxygenase 1(HO-1) via the Nrf2 pathway [47]. Through its anti-inflammatory and antioxidant qualities, quercetin lowers fat accumulation. It also controls intestinal microbiota, which can stop NAFLD. Data from a clinical trial showed that quercetin-rich onions significantly reduced the visceral fat area in subjects and improved liver function [48]. However, the limited administration, low bioavailability, and unstable nature of quercetin have limited application in NAFLD, and more advancements are necessary.

#### 3.1.2. Naringenin

Naringenin is mainly derived from the kernel shell of the fruit of the pedunculate tree (*Amacardi-um occidentale* L.), family Lacertidae. Additionally, naringin is a flavanoid found in oranges, grapes, and other vegetables and fruits that have been shown to have anticancer, anti-inflammatory, antioxidant, and other pharmacological properties [44,49]. It has been shown that naringenin reduces methionine choline diet-induced NAFLD in mice through its anti-inflammatory properties by suppressinginterleukin-1β (IL-1β) expression and downregulating the NLRP3/NF-κB pathway in KC and hepatocytes, thereby attenuating the inflammatory response in the liver [50]. A study has indicated that naringenin can activate AMPK, which leads to increased glucose uptake, decreased ATP content, and increased mitochondrial biogenesis in the myotubes of 3T3-L1 adipocytes and C2C12 myogenic cells. Through direct or indirect activation of the AMPK pathway, naringenin can mitigate NAFLD by increasing energy expenditure and regulating autophagy [51]. Furthermore, naringenin reduced hepatic lipid accumulation by significantly reducing the mRNA expression levels of monocyte chemotactic protein-1 (MCP1/Ccl2) and interleukin-6 (IL-6/II-6), leading to reduced hepatic lipid accumulation and decreased metabolic disorders associated with ovariectomy in female mice, including NAFLD [52]. Naringin can reduce the production of plasma fatty acids and the expression of liver pro-inflammatory mediators, including TNF-α, IL-1β, inducible nitric oxide synthase, matrix metalloproteinase (MMP-2,9), etc. Naringin has been demonstrated to reduce protein and lipid oxidation and improve antioxidant defense [53]. Clinical trials have demonstrated that NAFLD patients who received naringenin intervention experienced significant reductions in the indices of total cholesterol/high-density lipoprotein cholesterol (HDL-C), triglycerides/HDL-C, and low-density lipoprotein cholesterol/HDL-C [54]. The anti-inflammatory effects of naringenin may reduce the inflammatory process in the liver, improve metabolic function, and inhibit liver steatosis with minimal systemic side effects. Given the promising preclinical results, it is certainly worthwhile to conduct further clinical studies.

#### 3.1.3. Silymarin

Silymarin is a natural active substance obtained from the dried fruit of the plant Silymarin, family Asteraceae, which is an antioxidant and anti-inflammatory and regulates cell metabolism. It is used to treat various diseases [55]. Silymarin is used to treat NAFLD and other endoplasmic reticulum stress disorders by lowering the number of ER stress-related proteins such as glucose-regulating protein 78 kDa (GRP78) and X-box-binding protein-1 (XBP-1) [56]. In a model of fructose-induced non-alcoholic fatty liver disease in rats, silymarin reduced lipid index parameters such as aspartate aminotransferase (AST), aspartate aminotransferase (ALT), superoxide dismutase/hydrogen peroxidase (SOD/CAT), and thiobarbituric acid reactive substances (TBARS). It also decreased acetyl coenzyme A carboxylase-1 (ACC-1) and fatty acid synthase (FAS) protein expression levels, increased mRNA levels, and inhibited hepatic citrate synthase activity and lipogenesis, and improved dyslipidemia, oxidative stress, and steatosis [57,58]. In a clinical trial examining silymarin’s efficacy in NAFLD patients, the aspartate convertase to platelet ratio index was significantly reduced. These findings suggest that silymarin could be used to treat NAFLD by reducing liver fibrosis and that it is both safe and tolerable [59]. A clinical trial showed that silymarin when combined with vitamin C, vitamin E, coenzyme Q10 and selenomethionine can be effective in treating NAFLD. The trial showed significant reductions in ALT and AST, which are markers of liver function, and the preparation was well tolerated during administration, significantly improving liver dysfunction in patients with NAFLD [60]. Silymarin has been shown to have antioxidant properties and can effectively remove reactive oxygen species and prevent lipid peroxidation. It is also considered to be a safe and low-toxicity agent. Despite its promising properties, silymarin is not commonly used in clinical settings, and further patient trials are necessary to validate its potential benefits.

#### 3.1.4. Rutin

Rutin is a kind of flavonol that is found in a large number of plants, including passionflower, tea, and apples. It is also an important nutrient found in food. Rutin is mainly extracted from acacia rice [61]. Rutin plays a crucial role in regulating lipid metabolism and protecting cells from oxidative damage. The peroxisome proliferator-activated receptor alpha (PPAR-α) is a key controller of lipogenesis. By inhibiting free fatty acid synthesis through PPAR-α and its downstream targets, it may be possible to treat NAFLD [62]. It has been shown that rutin may have an adjuvant effect on the treatment of NAFLD [63,64]. However, more trials are required to confirm the therapeutic properties of rutin in a clinical setting and to determine the most effective way to administer it orally.

#### 3.1.5. Kaempferol

Kaempferol is a low-molecular-weight flavonoid that can be found in several food plants, including beans, kale, strawberries, and grapes. It is mainly derived from the rhizome of the ginger family, Kaempferia. Kaempferol has numerous pharmacological properties, including antioxidant, anti-inflammatory, and antibacterial properties [65]. Kaempferol was found to decrease the expression of several proteins involved in lipid metabolism and synthesis such as SREBP1, FAS, SOD-1, and two adipogenesis-related proteins, PPAR and C/EBPβ. The study also revealed that kaempferol effectively reduced lipid accumulation and oxidative stress caused by oleic acid in HepG2 cells, leading to a significant improvement in NAFLD [66]. A previous study investigated the therapeutic effects of kaempferol on mice with high-fat-diet-induced NASH using transcriptomic and metabolomic approaches. The results showed that kaempferol significantly reduced serum levels of ALT, low-density lipoprotein cholesterol (LDL-C) and total serum cholesterol (TC). This mechanism plays a potential therapeutic role with 277 genes including Cyp2b9, Cyp4a12b, Mup17, Mup7, and Mup16 [67]. Under certain conditions, kaempferol can have adverse effects. There is evidence that it reduces the oral availability of kaempferol when it reacts with iron [68]. Further clinical trials and in vivo studies are necessary to gain a more comprehensive understanding of the dosage effects of kaempferol and to assess its potential as a novel medication (Table 1).

#### 3.1.6. Anthocyanins

Anthocyanins, an important branch of flavonoids, are widely found in the flowers, fruits, seeds, and leaves of plants. Grape seeds are currently the main source of anthocyanins. Anthocyanins play a significant role in the prevention and treatment of many chronic diseases because of their anti-inflammatory, antioxidant, and anti-mutagenic capabilities [69]. Studies have shown that anthocyanins can decrease glycerol-3-phosphate acyltransferase 1 (GPAT1) activity by preventing mtGTAP1 from moving from the endoplasmic reticulum to the outer mitochondrial membrane. They can also increase protein kinase C phosphorylation and membrane translocation. Furthermore, studies in vivo showed that anthocyanins significantly improved high-glucose-induced diabetes and fatty liver disease by reducing hepatic mtGTAP1 activity [70]. Clinical experiments have shown that the activation of the NLRP3 inflammasome is highly increased in NAFLD patients, and anthocyanins can significantly reduce NLRP3 inflammasome components such as caspase-1, IL-1β, and IL-18, providing a basis for the development of anti-inflammatory therapy for NAFLD [71].

**Table 1:** Mechanisms of flavonoids in the treatment of NAFLD.

Natural Compound	Model	Function	Mechanism/Target	Reference
Quercetin	In vitro: FFA-induced model of lipid accumulation in HepG2 cells	Antioxidant	DNL pathway, SREBP-1, XBP-1	[40]
		Inhibits lipid accumulation in the liver	SREBP1c, FAS	[44]
	In vivo: T2MD-induced NAFLD and quercetin therapy models	Antioxidant Anti-inflammatory	FXR1/TGR5 pathway, IL-1β, TNF-α	[41]
	In vivo: HFD-induced mouse NAFLD models	Regulates the gut microbiota	TLR-4 pathway	[42,43]
		Inhibits hepatic iron ptosis	GPX4, COX-2, ACSL4	[46]
		Antioxidant	Nrf-2 pathway, HO-1	[47]
Naringenin	In vivo: MCD-induced mouse NAFLD models	Anti-inflammatory	NLRP3/NF-κB,IL-1β	[50]
	In vivo: HFD-induced mouse NAFLD models		CaMKKβ/AMPK/ACC pathway	[51]
	In vivo: a mouse model of NAFLD with ovarian removal	Regulates metabolic disorders	MCP1/Ccl2, IL-6	[52]
Silymarin	In vivo: fructose-induced mouse models of NAFLD	Effects of ER stress Antioxidant	GRP78, XBP-1 ACC-1,FAS	[56,57,58]
Rutin	In vitro: oleic-acid-induced NAFLD model of HepG2 cells	Oxidative stress regulates lipid metabolism	PPAR-α	[62]
	In vivo: fructose-induced mouse models of NAFLD	Oxidative stress, Anti-inflammatory	Caspase-3	[63,64]
Kaempferol	In vitro: oleic-acid-induced NAFLD model of HepG2 cells	Oxidative stress inhibits lipid accumulation	SREBP1,FAS,SOD-1	[66]
	In vivo: HFD-induced MASH mouse models	Inhibits lipid accumulation	Cyp2b9,Cyp4a12b,Mup17,Mup7,Mup16	[67]
Anthocyanins	In vivo: a mouse model of high-glucose-induced NAFLD	Oxidative stress	GPAT1	[70]

### 3.2. Alkaloids

Alkaloids are usually colorless, bitter-tasting nitrogen oxides that are found in the plant kingdom, especially in seeds [72]. Due to their wide range of pharmacological properties, including analgesic, anti-inflammatory, anti-tumor, antioxidant, and antibacterial effects, alkaloids are valuable sources for drug development [73]. Alkaloids improve NAFLD mainly through their anti-inflammatory and antioxidant effects, and inhibiting steatosis of the liver.

#### 3.2.1. Berberine

Berberine is an isoquinoline alkaloid that is extracted from Phellodendron and other Berberis plants. It has a variety of pharmacological effects on several organs, including anti-infective, anti-inflammatory, and anti-tumor properties [74]. There is no doubt that inflammation plays a key role in the development of NAFLD. Berberine can inhibit the expression of pro-inflammatory cytokines such as TNF-α, IL-6, IL-1β and monocyte chemoattractant protein-1 (MCP-1) through its anti-inflammatory effect. It has been shown that berberine can effectively treat NAFLD caused by palmitic acid and lipopolysaccharides by inhibiting the activation of endoplasmic reticulum stress and the expression of pro-inflammatory factors in hepatocytes [75]. Reduced expression of stearyl coenzyme A desaturase (SCD1) will lower lipid accumulation in over-nourished liver cells. It has been found that berberine induces AMPK phosphorylation, increases SREBP1c phosphorylation, inhibits SREBP1c nuclear translocation, reduces the binding of SREBP1c and SRE motif to the SCD-1 promoter, and reduces SCD-1 expression. This process reduces lipid accumulation by inhibiting the AMPK-SREBP1c-SCD-1 pathway [76]. Berberine has been found to be effective in treating NAFLD by improving glucolipid metabolism. In a study conducted on an NAFLD model produced by a high-fat diet, berberine was observed to decrease the occurrence of glucose, gluconeogenesis and adipogenesis. The mechanism of action involves inhibiting sugar production, regulating fat metabolism, and significantly reducing liver fat production [77]. Studies have shown that berberine can effectively treat NAFLD by reducing the expression of chemokine-like receptor 1 (CMKLR1) and CC-chemokine receptor-2 (CCR2) mRNA. This is achieved through the reduction of chemerin, its receptor, and by restoring the ratio of regulatory T cells to helper T cells (Treg/Th17) [78]. Berberine can also inhibit the expression of C/EBP homologous protein (CHOP), glucose-regulated protein 78 (Grp78), activating transcription factor 6 (ATF6), and other proteins by relieving endoplasmic reticulum stress in hepatocytes and regulating intestinal microbes, thus controlling liver metabolic disorder caused by liver injury [79]. Additionally, berberine red, which is the main metabolite of berberine, is also useful in the treatment of NAFLD. Berberine red can reduce adipose triglyceride lipase (ATGL), glucose kinase (GK) and PPAR-α, carnitine palmitoyltransferase-1 (CPT-1), ACC1, FAS and leukocyte differentiation antigen 36 (CD36), which are all factors that contribute to lipid accumulation [80]. The berberine compound has a protective effect on rats with HFD-induced fatty liver disease. According to this model, berberine can significantly reduce serum endotoxin and TNF-α levels and regulate intestinal microflora imbalances. Berberine intervention significantly reduced the level of Escherichia coli and Przewalski Lou Gehrig [81]. In vivo experiments have shown that berberine can inhibit liver fat deformation and significantly reduce liver TG, ALT, AST, and other markers. The mechanism is that berberine reverses the abnormal expression of microsomal triglyceride transfer protein (MTTP) and low-density lipoprotein receptor (LDLR) [82]. According to previous studies, berberine inhibits lipid accumulation by activating the sirtuin 1-Forkhead box O1–transcription factor sterol regulatory element binding protein 2 (SIRT1-FoxO1-SREBP2) pathway and alleviating the fatty degeneration of HepG2 cells caused by free fatty acids (FFAs) [83]. Berberine can also reduce the expression of uncoupling protein-2 (UCP2) and UCP2 mRNA in liver tissue, improve hepatocyte steatosis, and improve lipid metabolism disorders [84]. According to clinical research, berberine acts primarily through oral absorption, liver metabolism, and urine excretion [85]. However, most of the data on berberine’s use in treating NAFLD comes from preclinical studies, which highlights the need for more clinical trials to ensure the safety and tolerability of berberine in the future.

#### 3.2.2. Betaine

Betaine, also known as trimethylglycine, is a safe and stable natural chemical that is mainly sourced from the molasses of sugar beets. Both non-alcoholic and alcoholic fatty liver disease can be effectively treated using its anti-inflammatory effects [86]. Betaine can successfully improve NAFLD induced by fructose in mice. The mechanism is based on the fact that betaine can upregulate the expression of hepatic X receptor alpha (LXRα) and PPAR-α and reduces endoplasmic reticulum stress, inhibiting hepatic adipogenesis and NF-κB/NLRP3 inflammatory vesicle activation-mediated inflammation through its anti-inflammatory effects [87]. Abnormal DNA methylation is believed to contribute to abnormal liver gene expression, playing a role in the pathogenesis of NAFLD. Betaine has been shown to lower FAS and fatty acyl-coenzyme A oxidase (ACOX) mRNA expression, increased PPAR and microsomal triglyceride transfer protein (MTTP) mRNA expression, and greatly restore methylation levels. While many animal experiments have demonstrated betaine’s effectiveness in improving NAFLD, clinical trials have been marred by numerous errors [86]. Studies have demonstrated that betaine reduces the synthesis of active oxygen and nitrogen in the liver by increasing the content of glutathione and the activity of antioxidant enzymes, such as superoxide dismutase, catalase, and glutathione peroxidase, in a mouse model of NAFLD induced by a methionine choline deficiency diet (MCD). The mechanism is a decrease in the hepatic expression of the pro-inflammatory cytokines TNF-α and IL-6 and the pro-apoptotic mediator Bax, as well as a decrease in interleukin-10 (IL-10) and anti-apoptotic Bcl-2 levels, also associated with increased cytoprotective Akt/mTOR signaling and autophagy [88]. As a result, future research should focus on animal and clinical trials to eliminate various sources of experimental errors and ensure the therapeutic efficacy of betaine.

#### 3.2.3. Conophylline

Conophylline is a vinca alkaloid with a molecular weight of 794 and is a dimer of an aspidosperma-type indole alkaloid [89]. It has various biological activities such as antitumor, anti-inflammatory and antibacterial properties, among others [90,91]. Methionine-choline-deficient diet (MCD) can induce not only steatohepatitis, but also adipototissue degradation [92,93]. Studies have shown that conophylline can improve type 2 diabetes and inhibit chemically induced cirrhosis, thereby effectively improving NAFLD [90]. Its mechanism for treating NAFLD has also been linked to reduced levels of PPARα mRNA, a peroxide in the liver [94]. On the downside, conophylline is less effective at inhibiting liver fibrosis because conophylline cannot participate in the fibrinolytic process [95].

#### 3.2.4. Oxymatrine

Oxymatrine is a quinolizidine alkaloid extracted from bitter ginseng, and it exhibits a variety of pharmacological effects, including anti-inflammatory, antiviral, and antioxidant properties [32]. In a conductivity analysis, oxymatrine significantly reduced steatosis, decreased FASN and SCD1 expression, and increased SIRT1 expression and AMPK phosphorylation [96]. Furthermore, oxymatrine decreased FAS enzymatic activity and increased CPT1A enzymatic activity in mice with NAFLD induced by high glucose, inhibiting hepatic lipid synthesis [97]. In clinical trials, oxymatrine has shown significant effects when administered intravenously at doses of 1000–1500 mg/day [98]. Consequently, oxymatrine has been considered as a potential treatment option for NAFLD in recent years.

#### 3.2.5. Ramulus Mori (Sangzhi) Alkaloids

Ramulus Mori (Sangzhi) alkaloids (SZ-A) are a group of polyhydroxy alkaloid active ingredients isolated from traditional Chinese medicine. While they have been shown to have good hypoglycemic effects, they have also been found to reduce obesity and non-alcoholic fatty liver disease induced by a high-fat diet, as well as improve obesity-related adipose tissue metabolism and inflammation. Studies have shown that SZ-A can regulate the gut microbiota of obese mice and change the composition of metabolites. In addition, SZ-A enriched the number of goblet cells and reduced inflammatory colon injury and pro-inflammatory macrophage infiltration induced by a high-fat diet in obese mice, reducing the symptoms of NAFLD to some extent [99] (Table 2).

### 3.3. Phenolic Compounds

Phenolic compounds are naturally occurring active biomolecules found primarily in plants and have a wide range of biological activities, including antioxidant, anti-inflammatory, and anti-proliferative effects [100]. The treatment of NAFLD with phenolic compounds mainly involves inhibiting liver fat deformation and antioxidant effects.

#### 3.3.1. Curcumin

Curcumin is a polyphenolic compound in turmeric that has many pharmacological effects, including antioxidant, anti-inflammatory, anti-proliferative, and anti-angiogenic effects [101]. Studies have shown that curcumin can protect against the development of non-alcoholic fatty liver disease (NAFLD) caused by high fat and high fructose in mice. The mechanism is based on the fact that curcumin reverses the expression of cytochrome P4503A (CYP3A) and cytochrome P4507A (CYP7A) by regulating the LXR pathway, and reduces the expression of CD36, SREBP1c, and small heterodimer partner (SHP) by regulating the FAS and Nrf2 pathways, thereby lowering hepatic steatosis [102]. According to an in vitro study, curcumin reduces fat buildup in the liver by preventing the distribution of citrate in the AMPK pathway, which controls the dysregulation of SLC13A5/ACLY expression. This prevents citrate transport and metabolism [103]. Furthermore, curcumin has been shown to prevent hepatic steatosis by promoting the phosphorylation of hepatic activator of transcription 3 (STAT3) and by suppressing the expression of SREBP1c and suppressor of cytokine signaling 3 (SOCS-3). This helps to regulate lipid metabolism and reduce the accumulation of fat in the liver [104]. In NAFLD mice, curcumin has been shown to improve intestinal barrier dysfunction in NAFLD mice by upregulating the expression of the tight junction protein-small band occludin 1. It also decreased the expression of myeloid differentiation factor 88 (MyD88), and inhibited p65 nuclear translocation and NF-κB DNA-binding activity in the liver, which in turn suppressed hepatic steatosis [105]. PPAR-α gene methylation plays a role in the pathophysiology of NAFLD. A previous study showed that curcumin greatly decreased methylation levels, enhanced PPAR-α protein expression, and significantly reduced lipid accumulation in NAFLD rats [106]. Curcumin has also been found to increase the expression of phosphorylated serine-threonine protein kinase (pAKT) and P13K protein, protecting LO2 cells from oleic acid (OA)-induced NAFLD, improving glucose uptake in hepatocytes, with curcumin also reducing NO and ROS levels via Nrf2 signaling [107]. Based on the results of the phase I clinical trials, curcumin is also safe for humans at high doses (12 g/day). However, due to poor absorption and rapid metabolism, its bioavailability is low [101]. In order to make it a breakthrough drug with improved bioavailability, research is currently underway regarding its bioavailability.

#### 3.3.2. Epigallocatechin-3-Gallate

Epigallocatechin-3-gallate (EGCG) is the most abundant and potent catechin in green tea. It processes anti-tumor, antioxidant, anti-inflammatory, and immunomodulatory properties [108]. A study has found that EGCG may alleviate NAFLD by reducing apoptosis and promoting autophagy through a pathway related to reactive oxygen species and mitogen-activated protein kinase (ROS/MAPK) [109]. Fibroblast growth factor-21 (FGF21) is a metabolically active hormone that contributes to lipid metabolism and is associated with the pathogenesis of NAFLD. By increasing Nrf2 levels in NAFLD, EGCG reduces lipid accumulation, reducing oxidative stress. Additionally, EGCG increases FGFR/AMPK expression by reducing FGF21 resistance, thereby reducing hepatocyte injury and dysfunction [2]. Several clinical studies have shown that EGCG is effective in reducing body weight, oxidative stress, and liver injury markers, and in restoring the metabolism of lipids when taken in a dose-dependent manner [110]. EGCG has a greater safety profile and is less toxic However, the optimal dosage for treating NAFLD is still unclear, and there is a need for further research in this area.

#### 3.3.3. Resveratrol

Resveratrol is a phenolic compound from the stilbene family of phenols, with a C6-C2-C6 structure that is found abundantly in plants such as cassia seeds, grape shells, and white tea. It is mainly derived from the rhizome extract of Polygonum multiflorum. Resveratrol has powerful antioxidant, anticancer, and anti-inflammatory functions [111]. Many studies have shown that resveratrol activates the sirtuins pathway (STRT1) to treat NAFLD. A major mechanism by which resveratrol reduces fat accumulation is through activation of the STRT1-FOXO1 pathway, which inhibits the acetylation of SREBPE-1c to reduce metabolic disorders [112,113]. Furthermore, a decrease in STRT1 in the hepatocytes can result in the inflammation of the liver [114]. When 3T3-L1 cells are stimulated with TNF-α, siRNA-mediated SIRT1 induction increase the expression of cytokine mRNA [115]. In the meantime, increased SIRT1 expression inhibits the production of pro-inflammatory cytokines like NF-κB and TNF-α, preventing damage to the liver’s metabolism cause by a high-fat diet [116]. Studies have shown that resveratrol can reduce OA-induced L02 cell apoptosis, mitochondrial dysfunction, and ROS production. It can also treat NAFLD by reducing the expression of caspase-3 and p53, and increasing the expression of B-cell lymphoma 2 (Bcl-2), thereby reducing hepatic lipotoxicity [117]. Several clinical trials have shown that resveratrol can be safety used as a dietary supplement at an acceptable daily intake of 450 mg/day. Additionally, some trials have shown that 5000 mg of resveratrol per day has no adverse effects [118]. Resveratrol has been shown to be safe and tolerable, making it a promising candidate for the treatment of NAFLD. However, further large-scale clinical trials are necessary to substantiate its effectiveness.

#### 3.3.4. Caffeic Acid

Caffeic acid is an important phytonutrient that is present in various plants, fruits, and vegetables. It exhibits a broad spectrum of functions, including antioxidant, antibacterial, anti-inflammatory, and immunomodulatory properties [119]. Several studies have demonstrated the effective treatment of NAFLD with caffeic acid. It has been found to improve the condition of NAFLD by regulating dysbiosis of the intestinal microflora, reducing LPS-mediated inflammation, and inhibiting the expression of lipid metabolism genes [120]. Caffeic acid has been shown to reduce lipid accumulation and adipogenic marker levels by reducing ER stress and enhancing autophagic indicators. This mechanisms linked to elevated TG levels, ER stress, and insulin resistance [121]. Another study found that caffeic acid significantly reduced the production of TG and cholesterol by upregulating the phosphorylation of the downstream target enzyme acetyl coenzyme A carboxylase (ACC) through the AMPK pathway, while downregulating the expression of SREBP1, FAS, glycerol-3-phosphate acyltransferase (GPAT), and 3-hydroxy-3-methyl glutamate coenzyme A reductase (HMGCR) [122]. Despite its potential therapeutic benefits, the low oral utilization of caffeic acid is a significant challenge. Caffeic acid is present in esterified foods, where only a small fraction is absorbed during the ingestion process. Therefore, further research is needed to address this issue and improve the bioavailability of caffeic acid for its effective use in the treatment of NAFLD and other related conditions [123].

#### 3.3.5. Gastrodin

Gastrodin is a bioactive substance that can be isolated from the rhizome of Gastrodia aspera. It has various beneficial effects on the central nervous system and can be used to treat neurological illnesses like epilepsy, depression, and anxiety [124]. Recently, the use of gastrodin for the treatment of fatty liver disease has been covered in some recent publications. According to an in vivo study, it was found that gastrodin significantly reduces liver fibrosis by activating AMPKα. This mechanism prevents the synthesis of lipotoxic chemicals, subsequently lowering the release of profibrotic cytokines and the inflammatory response [125]. Another in vivo study also showed that gastrodin can inhibit liver steatosis by activating AMPK and improving lipid metabolism. Furthermore, it can also improve liver oxidative stress and inflammatory response by activating the Nrf2 pathway [126]. Due to its long history of safe use, gastrodin has the potential to become a new drug for the treatment of NAFLD in the future. However, further clinical trials are required to establish its effectiveness and safety (Table 3).

### 3.4. Saponins

Numerous natural plants contain saponins, which possess anti-inflammatory, antioxidant, hypocholesterolemic, and hepatoprotective effects. Saponins offer several health benefits and can be utilized to treat NAFLD [127]. The treatment of NAFLD with saponins primarily occurs through their antioxidant stress effects.

#### 3.4.1. Panax Notoginseng Saponins

*Panax Notoginseng* Saponins (PNS) are among the primary active ingredients found in Panax notoginseng root, which is useful in cardiovascular protection, neuroprotection, gastrointestinal protection, liver protection, etc. [128]. The activation ofhepatic stellate cells and liver fibrosis are among the mechanisms leading to the development of NAFLD [129]. Researchers have found that PNS can inhibit the activation of hepatic stellate cells and block NF-κB and MAPK signaling. Furthermore, PNS can reduce PPAR-α expression induced by carbon tetrachloride and coll-a1 expression levels in liver tissue, thereby reducing liver fibrosis [130]. Another study discovered that PNS improved the dysfunction of lipid metabolism in hepatocytes induced by fatty acids via Toll-like receptor 4 (TLR4) and AMPKα signaling [131]. Clinical studies revealed that consuming PNS resulted in mild side effects such as subcutaneous bleeding, fecal occult blood, and skin rash [132]. However, further clinical trials are necessary to confirm its safety.

#### 3.4.2. Saikosaponin

Saikosaponin is the main active ingredient in Chai Hu, In vitro and in vivo, it exhibits anti-inflammatory, anti-tumor, antiviral, and liver protection properties [133]. Saikosaponin can significantly reduce the amount of lipids in adipose tissue. Its mechanism of action involves being an effective PPAR-α Activator, which can activate PPAR-α, Promote the oxidation of fatty acid (FA) and induce the expression of anchor protein insulin-induced gene (INSIG1/2), thus inhibiting the expression of SREBP1 and the synthesis of FA [71]. Another study found that saikosaponin could downregulate the expression of fatty acid synthase (FASN) and ACACA (acetyl coenzyme A carboxylase) to reduce FA biosynthesis and induce carnitine palmitoyltransferase-1 (CPT-1), acyl-coenzyme A oxidase 1 (ACOX1) expression to promote FA degradation, thereby reducing fat accumulation [134]. Bupleurum has a hepatoprotective effect, and several studies have explored the potential of its active ingredient, saikosaponin, in treating NAFLD. In addition, toxicological studies have indicated that high doses of saikosaponin may cause nephrotoxicity in humans [133]. Therefore, additional clinical trials are needed to determine its safe dosage.

#### 3.4.3. Ginsenoside Rg1

The natural pharmaceutical ingredient ginsenoside Rg1 is derived from Ginseng and has pharmacological effects such as anti-aging, anti-inflammatory, and oxidative stress [135]. Recent studies have shown that ginsenoside Rg1 exhibits strong antioxidant properties and can be used in the treatment NAFLD by minimizing liver damage caused by oxidative stress [136]. Bioinformatics analysis has showen that Ginsenoside Rg1 can effectively decrease liver fat accumulation, promote liver glycogen synthesis, reduce oxidative stress-induced liver damage, and regulate liver cell metabolism through the ECM receptor and PI3K-AKT pathway, Key proteins such as EGFR and STRT1 may be involved in these processes [135]. Ginsenoside Rg1 can prevent the increase in phosphorylation of FOXO1 in NAFLD, increase the levels of the antioxidant enzymes SOD and CAT, and maintain the activity of FOXO1 in the liver. These effects enhance the antioxidant capacity of Ginsenoside Rg1, promote metabolic homeostasis, and protect the liver from aging-related liver diseases like NAFLD [137]. In addition to its antioxidant properties, Ginsenoside Rg1 has also been found to increase the expression of PPAR-α, which enhances the metabolism of FFA and TG while reducing ER stress. The main proteins in this process are cysteine-containing aspartate-specific protease 12 (Caspase-12) and GRP78. Additionally, Ginsenoside Rg1 reduces the production of inflammatory cytokines like interleukin 1 (IL-1) and interleukin 18 (IL-18), thereby alleviating NAFLD through its anti-inflammatory function [138]. Several studies have shown that the anti-inflammatory activities of Ginsenoside Rg1 can also prevent the development of NAFLD. Ginsenoside Rg1 improves hepatocyte morphology, reduces hepatic lipid accumulation, reduces the hepatic inflammatory response, and lessens liver degeneration through the AMPK-NF-κB pathway, thereby reducing liver injury. Ginsenoside Rg1 has also been found to decreases the release of cytokines such as IL-1, IL-6, IL-18, and TNF-α [139,140]. However, it should be noted that Ginsenoside Rg1 has limited oral bioavailability because the intestinal mucosa has a low mucosal permeability [141]. Therefore, a large number of clinical studies are needed to further verify its effectiveness.

#### 3.4.4. Diosgenin

Diosgenin is a steroidal saponin that is widely present in nature. It has anti-inflammatory and antioxidant properties, making it useful for the treatment of various diseases. In addition, it can also play a key therapeutic role in the treatment of liver fat diseases by acting as a key regulator in lipid metabolism [142]. Studies have shown that diosgenin can inhibit the accumulation of fatty acids and triglycerides in the liver by suppressing the SREBP-1c and FASN genes, thereby improving histopathological fat changes [143]. In vitro studies have also shown that diosgenin can significantly ameliorate palmitic-acid-induced steatosis in LO2 cells by activating the AMPK/ACC/CPT-1A pathway and inhibiting the SREBP-1c/FAS pathway [144]. However, a large number of clinical trials are required to determine the safety and efficacy of diosgenin for the treatment of NAFLD (Table 4).

### 3.5. Plant Polysaccharides

Plant polysaccharides are active components that help maintain living organisms. They consist of many identical or different monosaccharides, linked together through α or β glycosidic bonds. These compounds are commonly found in natural plants, including starch, cellulose, polysaccharide and pectin [145]. Plant polysaccharides have biological activities, including immune regulation, anti-tumor, hypoglycemic, and liver-protective properties.

#### 3.5.1. *Lycium barbarum* Polysaccharides

*Lycium barbarum* polysaccharides are one of the main components of *lycium* barbarum, and as heteropolysaccharides, they have the effects of lowering blood sugar and blood lipids [146]. *Lycium barbarum* polysaccharides have been found to be effective in treating NAFLD by reducing inflammation and steatosis in the liver. Studies have shown that SIRT1 plays an important role in regulating lipid metabolism in liver cells. *Lycium barbarum* polysaccharides can promote AMPK phosphorylation through SIRT1-dependent signaling, while activating the STRT1/AMPK pathway to reduce FAS expression, thereby achieving the goal of inhibiting liver steatosis [147]. An in vivo study has shown that *lycium barbarum* polysaccharides can reduce NF-κB activity to alleviate liver inflammation and liver injury [148]. Moreover, clinical trials also showed that *lycium barbarum* polysaccharides can regulate the human gut microbiota to improve NAFLD, providing a promising basis for the treatment of NAFLD by *lycium barbarum* polysaccharides in the future [134].

#### 3.5.2. *Schisandra chinensis* Polysaccharides

*Schisandra chinensis* polysaccharides are the main active ingredient in *Schisandra chinensis*. *Schisandra chinensis* is mostly used for liver protection in clinical practice, with obvious therapeutic effect. *Schisandra chinensis* can improve NAFLD by reducing lipid accumulation. Some studies have shown that *Schisandra chinensis* polysaccharides can reduce the expression of FAS, SREBP-1c and LXRα in mice with t NAFLD induced by HFD, reducing the levels of TC and TG in the body, alleviating liver cell steatosis and necrosis [149]. Moreover, some studies have shown that Schisandra chinensis polysaccharides also have a certain protective effect against liver injury. By downregulating the expression of Nrf2 and HO-1, this can significantly reduce immune liver injury [150]. The metabonomic enrichment analysis showed that the therapeutic pathways of *Schisandra chinensis* polysaccharide in NAFLD mainly included the metabolism of ascorbic acid and uronic acid, the mutual transformation of pentose and glucuronic acid, the metabolism of nicotinic acid and nicotinamide, the citric acid cycle, the metabolism of butyric acid, and the metabolism of inositol phosphate [151]. The research on *Schisandra chinensis* polysaccharide should be deepened to find the optimal extraction method, enhance chemical structure analysis, and advance the understanding of its mechanism for treating NAFLD. Moreover, increasing the number of clinical trials to demonstrate its efficacy is crucial.

#### 3.5.3. Astragalus Polysaccharides

Astragalus polysaccharides are a key active component isolated from Astragalus membranaceus. They have good regulatory effects on blood lipids and blood sugar, as well as antioxidant stress and antifibrosis effects [152]. A previous study showed that Astragalus polysaccharides can improve NAFLD by regulating the gut microbiota, by increasing the expression of AMPK and PPAR, thereby reducing their lipid accumulation. This is also related to the reduced expression of SREBP-1 [153]. Astragalus polysaccharide also has a regulatory effect on metabolic disorder. Research shows that through metagenomic sequencing and metabonomic analysis, Astragalus polysaccharide changes the pathway of glutathione metabolism and purine metabolism, and improves the symptoms of metabolic disorder by regulating the intestinal microbiota [154]. However, due to the low oral availability of Astragalus polysaccharides, further clinical trials are needed to support these findings (Table 5).

### 3.6. Other Compounds

Paeoniflorin is a monoterpene glucoside component with hepatoprotective, antioxidant and other pharmacological properties that play a crucial role in managing NAFLD. Its mechanism of hepatoprotection involves activating the AMPK pathway, which inhibits hepatic steatosis, stimulating β-oxidation and gluconeogenesis. Additionally, paeoniflorin regulates lipid metabolism, and improves fructose-induced hepatic steatosis and insulin resistance, further inhibiting steatosis [155,156]. It has been shown by pharmacokinetic studies that paeoniflorin is poorly absorbed by the body when administered orally, with only about 3–4% of the drug being absorbed after oral administration to rats [157]. Therefore, further research is needed to develop the therapeutic applications of paeoniflorin.

Andrographolide is a plant compound that exhibits anti-inflammatory activity and ameliorates NAFLD by inhibiting the NF-κB pathway. It has been shown that andrographolide reduces IL-1β protein expression by inhibiting the NF-κB pathway, thereby reducing the liver’s inflammatory response and liver fibrosis [158]. Additionally, andrographolide was demonstrated to treat NAFLD in an in vitro experiment by preventing steatosis through lower fatty acid uptake in oleic-acid-treated LO2 cells and downregulating the expression of fatty acid transport protein (FATP2) [159].

Ursolic acid is a secondary plant metabolite that is commonly found in fruit peels, stems, and leaves, which possesses anticancer and liver-protective properties [160]. Through in vivo experiments conducted on rats, researchers found that ursolic acid can reverse hepatic steatosis in the context of high-fat diets by improving the levels of key proteins involved in lipid metabolism through the PPAR-α pathway [161]. Liver X receptor alpha (LXRα) is a multifunctional nuclear receptor that regulates lipid homeostasis. Studies have demonstrated that ursolic acid decreases LXR response element and SREBP1c gene initiation and activity, which decreases liver steatosis and improves NAFLD [90]. However, studies have also reported that ursolic acid can cause tolerable toxicity and adverse effects such as nausea and diarrhea. Therefore, further investigation is necessary, including phase II clinical trials, to fully understand the efficacy and side effects of ursolic acid as a treatment for NAFLD [160].

Osthole, also known as osthol, is a coumarin derivative that is present in several medicinal plants such as Cnidium monnieri and Angelica pubescens. It has anti-inflammatory, anti-tumor, antibacterial, and other biological activities [162]. Several studies have shown that osthole can treat NAFLD by upregulating PPAR-α and downregulating liver proteins like NF-κB, SREBP1c, and FAS, which ultimately reduces liver steatosis and inflammation [144,163]. However, to support the therapeutic efficacy of osthole on NAFLD, more clinical evidence is needed, as there are only a limited number of clinical research trials available.

Lycopene, the most abundant carotenoid in tomatoes, contains 11 conjugated double bonds, making it a powerful antioxidant [164]. CYP2E1 is one of the microsomal cytochromes that produce ROS, and lycopene has been shown to minimize fatty infiltration in the liver. The mechanism is based on the fact that lycopene can reduce the generation of ROS by downregulating the expression of the proteins CYP2E1 and TNF-α [165]. Lycopene also has been found to have protective effects on HFD-induced NAFLD in mice. Lycopene ameliorated liver inflammation and fibrosis in a dose-dependent manner by reducing the mRNA expression levels of LPS, interferon-gamma (IFN-γ), TNF-α-induced macrophage M1 markers, and transforming growth factor β1 (TGF-β1)-induced fibrogenic expression in stellate cells, all of which can be attributed to its antioxidant properties [166]. A previous study found that lycopene inhibited stearic acid (SA) by downregulating fatty acid binding protein 7 (FABP7) through the normalization of miR-21 levels [167]. Lycopene may also prevent liver cell damage. Through the activation of the Nrf2-HO-1 pathway in hepatocytes, lycopene promotes the transfer of Nrf2 from the cytoplasm to the nucleus, ultimately protecting the liver cells [168]. Lycopene reduces α7 nicotinic acetylcholine receptor expression in the lung and NF-κB and CYP2E1 expression in the liver in a tobacco-carcinogen-induced ferret model of NAFLD, resulting in ameliorative effects on hepatocyte injury [169]. In addition, co-administering calcium supplements significantly reduces lycopene’s bioavailability by 84% [170]. In recent years, lycopene has emerged as a potentially effective treatment for NAFLD, although further clinical trials are needed to assess its adverse effects and pharmacokinetic profile (Table 6).

## 4. Discussion

Non-alcoholic fatty liver disease (NAFLD) is a prevalent chronic liver disease worldwide, and its incidence is increasing every year. Unfortunately, there is no definitive drug available to halt its progression, and the existing drugs with therapeutic effects have limitations. Despite the scientific community’s efforts, early diagnostic methods are limited due to the benign and reversible nature of the disease, which hinders the successful creation of treatments. Although diet and exercise are well-known methods to improve NAFLD, their effectiveness and applicability are limited. However, several natural substances have been extensively studied and have been proven to have beneficial pharmacological effects on NAFLD.

This paper provides a summary of natural monomeric compounds derived from plants that have shown promise in treating NAFLD. Although some of these compounds have been studied for their mechanism of action, their clinical use is limited due to the absence of clinical trials. Compounds such as naringenin, silymarin, anthocyanin, betaine, and serpentine have not undergone clinical studies, but may hold potential for the treatment of NAFLD and require further research to confirm their effectiveness. Various natural compounds, including rutin, kaempferol, lycopene, quercetin, caffeic acid, curcumin, ginsenoside Rg1, and paeoniflorin, have been found to have low oral bioavailability. While these compounds have shown promise in treating non-alcoholic fatty liver disease (NAFLD), more research is needed to understand their mode of administration and specific effects. The substances berberine, catechin, panaxoside, chai hu saponin, and ursolic acid have been associated with adverse effects such as vomiting and diarrhea after administration, and further investigation is needed into their safety, resistance, and dosage. The low oral availability of natural compound components and the lack of clinical trials are contributing factors that hinder the development of effective drugs and require further proof from specialized researchers.

NAFLD is consistent with the “multiple-hit” theory and is caused by multiple factors (lipotoxicity, oxidative stress, inflammation, and dysbiosis of the intestinal flora). However, there is no conclusive evidence to suggest that these compounds are expressed through a single protein pathway or that multiple protein pathways are expressed in a coordinated manner to exert therapeutic effects. Some natural compounds may have modulatory effects on multiple pathways or targets. There is a need for in-depth research using various techniques, including metabolomics, transcriptomics, proteomics, and macro genetic sequencing, in this area of natural chemical research, which is becoming increasingly popular.

While diet and exercise are typically the first steps in treating NAFLD, pharmacological treatment can also be necessary. This paper examines the mechanisms and roles of natural compounds in the prevention and treatment of NAFLD, providing valuable information for the development of new drugs. These compounds have been shown to reduce oxidative stress, insulin resistance, and lipid accumulation in the liver, and to interact with hepatic lipid metabolism and inflammation. In order to improve the treatment of NAFLD, we should consider the combination of natural compounds with existing drugs. To further investigate this possibility, we recommend conducting larger, randomized clinical trials over a longer duration to determine the role of these compounds in the treatment of NAFLD.

This review provides an update on the potential benefits of some natural compounds in preventing and treating NAFLD, with the aim of providing new potential therapeutic lead compounds and references for innovative drug development and clinical treatment. Many natural compounds have shown potential in the treatment of NAFLD. Most current research is focused on compounds with antioxidant effects and anti-inflammatory powers, compounds that can reduce the symptoms of NAFLD. In the future, combining natural compounds with existing drugs may be possible to better protect the liver by exerting the antioxidant and anti-inflammatory abilities of natural compounds while inhibiting liver steatosis.

## 5. Methodology

In this review, we searched the PubMed database (https://pub-med.ncbi.nlm.nih.gov/ (accessed on 11 January 2023)) and Google Chrome (https:///scholar.Google.com/ (accessed on 11 January 2023)). In this methodology, the names of natural compounds are replaced with “NC”, and search terms include “(non-alcoholic fatty liver disease) OR (non-alcoholic fatty liver)”, “(non-alcoholic fatty liver disease) OR (non-alcoholic fatty liver) AND (NC)”, “(Clinical) AND (NC)”, and “(pharmacokinetics) AND (NC)”.

## 6. Conclusions

To summarize, this review highlights the potential of natural compounds to treat NAFLD. Natural compounds can treat diseases through multiple components, multiple targets, and multiple approaches. The natural compounds described in this article have therapeutic and beneficial effects on NAFLD, and can provide a reference for potential drugs in the future clinical treatment of NAFLD. However, when considering the development of natural compounds for the treatment of NAFLD, their safety issues cannot be ignored, and more experiments are therefore needed.

## Figures and Tables

**Table 2:** Mechanisms of alkaloids in the treatment of NAFLD.

Natural Compound	Model	Function	Mechanism/Target	Reference
Berberine	In vivo: OB/OB mouse liver	Reduces lipid accumulation in the liver	AMPK-SREBP1c-SCD-1	[76]
	In vivo: a mouse model of Tulemeisol-induced endoplasmic reticulum stress	Relieves endoplasmic reticulum stress	CHOP, Grp78, ATF6	[79]
	In vitro: oleic-acid-induced NAFLD model of HepG2 cells	Regulates glucose and lipid metabolism	ATGL,GK,PPARα,CPT-1,ACC1,FAS,CD36	[80]
	In vivo: HFD-induced rat models of NAFLD	Improve glycolipid metabolism	SCD-1	[76]
		Anti-inflammatory regulates the intestinal microbiota	TNF-α, IL-6, IL-1β, MCP-1	[81]
		Inhibits hepatic steatosis	MTTP, LDLR	[82]
	In vitro: FFA oleic-acid-induced model of NAFLD in HepG2 cells		SIRT1-FoxO1-SREBP2 pathway	[83]
Betaine	In vivo: fructose-induced mouse models of NAFLD	Anti-inflammatory Antioxidant	LXRα, PPARα, NF-κB/NLRP3	[87]
	In vivo: MCD-induced mouse models of NAFLD		TNF-α, Bax, IL-10, Bcl-2	[88]
Conophylline	In vivo: MCD-induced rat models of NAFLD	Inhibits cirrhosis	PPARα	[90]
Oxymatrine	In vivo: High-glucose-induced NAFLD model in mice	Inhibition of hepatic lipid synthesis	FAS	[97]
SZ-A	In vivo: HFD-induced C57 mice models of NAFLD	Regulates the gut microbiota		[99]

**Table 3:** Mechanisms of phenolic compounds in the treatment of NAFLD.

Natural Compound	Model	Function	Mechanism/Target	Reference
Curcumin	In vivo: HFHFr-induced mouse NAFLD models	Inhibits hepatic steatosis	LXRα-FAS-Nrf2 pathway,CYP3A,CYP7A,CD36,SREBP1c,SHP	[102]
	In vitro: palmitic-acid-induced NAFLD model of HepG2 cells		AMPK pathway, SLC13A5, ACLY	[103]
	In vivo: HFD-induced mouse NAFLD models		MyD88	[105]
	In vitro: OA-induced rat NAFLD models	Oxidative stress regulates lipid metabolism	pAKT, P13K	[107]
EGCG	In vivo: HFD-induced mouse NAFLD models	Reduces apoptosis and promotes autophagy	ROS/MAPK pathway	[109]
	In vitro: FFA-induced primary mouse hepatocytes	Oxidative stress	FGFR/AMPK pathway, FGF21, Nrf2	[2]
Resveratrol	In vitro: palmitic-acid-induced NAFLD model of HepG2 cells	Reduces fat accumulation	SIRT1	[112,113]
	In vivo: HFD-induced mouse NAFLD models	Regulates metabolic balance		[116]
	In vivo: OA-induced model of NAFLD in L02 cells	Antioxidants, reduce liver lipid toxicity	caspase-3, p53, Bcl-2	[117]
Caffeic acid	In vivo: HFD-induced mouse NAFLD models	Regulates intestinal flora Anti-inflammatory	FAS	[120]
		Reduces lipid accumulation	ER stress and autophagy	[121]
	In vitro: OA-induced models of NAFLD in HepG2 cells	Inhibits fat production	AMPK pathway, SREBP1, FAS, GPAT, HMGCR	[122]
Gastrodin	In vivo: HFD-induced mouse NAFLD models	inhibits liver steatosis	AMPKα	[125]
			Nrf2 pathway	[126]

**Table 4:** Mechanisms of saponins in the treatment of NAFLD.

Natural Compound	Model	Function	Mechanism/Target	Reference
*Panax Notoginseng* Saponins	In vivo: HFD-induced mouse NAFLD models	Anti-inflammatory Antioxidant Anti-fibrosis	MAPK, NF-κB pathway, PPAR-α, coll-a1	[130]
			CD14, TLR4	[131]
Saikosaponin	In vitro: NAFLD model of HepG2 cells	Reduces lipid accumulation	PPAR-α, INSIG1/2, SREBP-1	[71]
	In vivo: HFD-induced mouse NAFLD models		FASN, ACACA, ACOX1, CPT-1α	[134]
Ginsenoside Rg1	In vivo: D-galactose-induced mouse NAFLD models	Oxidative stress	FOXO1, SOD, CAT	[137]
	In vivo: HFD-induced mouse NAFLD models	Antioxidant Reduce ER stress Anti-inflammatory	PPAR-α, Caspase-12, GRP78, IL-1β, IL-18	[138]
	In vitro: palmitic-acid-induced DAFLD models of HepG2 cells	Anti-inflammatory	IL-1, IL-6, IL-18, TNF-α	[139]
			AMPK-NF-κB pathway	[140]
Diosgenin	In vivo: HFD-induced mouse NAFLD models	Inhibit the accumulation of fatty acids and triglycerides in the liver	SREBP-1c, FASN	[143]
	In vitro: LO2 cell NAFLD models induced by palmitic acid	Improve steatosis	AMPK/ACC/CPT-1A pathway, SREBP-1c/FAS pathway	[144]

**Table 5:** Mechanisms of plant polysaccharides in the treatment of NAFLD.

Natural Compound	Model	Function	Mechanism/Target	Reference
*Lycium barbarum* polysaccharides	In vivo: HFD-induced mouse NAFLD models	Inhibiting steatosis	STRT1/AMPK pathway	[147]
	In vivo: MCD-induced rat models of NAFLD	Anti-inflammatory	NF–κB	[148]
*Schisandra chinensis* polysaccharides	In vivo: HFD-induced mouse NAFLD models	Reducing lipid accumulation	FAS, SREBP-1c, LXRα	[149]
Astragalus polysaccharides	In vivo: HFD-induced mouse NAFLD models	Regulating the gut microbiota	PPARα, AMPK	[153]
		Improve metabolic disorders		[154]

**Table 6:** Mechanisms of other compounds in the treatment of NAFLD.

Natural Compound	Model	Function	Mechanism/Target	Reference
Paeoniflorin	In vivo: HFD-induced mouse NAFLD models	Regulates lipid metabolism	Insulin signaling pathway	[155]
	In vivo: a fructose-induced model of rat NAFLD	Inhibits hepatic steatosis, Anti-inflammatory	AMPK pathway	[156]
Andrographolide	In vivo: choline-deficient amino-acid-induced mouse NAFLD model		NF-κB, IL-1β	[158]
Ursolic acid	In vivo: HFD-induced rat NAFLD models		PPAR-α PPAR-α	[161]
Osthole	In vitro: lipopolysaccharide-induced hepatocytes			[144]
	In vivo: high-fat, high-sugar-induced model of rat NAFLD		SREBP1c, FAS	[163]
Lycopene	In vivo: HFD-induced rat models of NAFLD	Antioxidant	CYP2E1,TNF-α	[165]
			LPS,IFN-γ, TNF-α,TGF-β1	[166]
	In vitro: stearic-acid-induced Hepa1-6 cells	Regulates lipid metabolism in the liver	FABP7	[167]
	In vivo: a model of tobacco-carcinogen-induced NAFLD in ferrets	Anticancer	NF-κB, CYP2E1	[168]

## Data Availability

Not applicable.
